# Supplementary material for: Coconut Milk-Derived Bioactive Peptides as Multifunctional Agents Against Hyperglycemia, Oxidative Stress, and Glycation: An Integrated Experimental and Computational Study
Source: Int J Mol Sci. 2025 Dec 29;27(1):360. doi: 10.3390/ijms27010360 (PMC12785583; doi:10.3390/ijms27010360)
Supplement: Supplementary file 1 [file ijms-27-00360-s001.zip › ijms-4042730-supplementary.pdf]

Supplementary Materials

Table of Contents

Table S1. Outcomes of *insilico* analysis: Coconut milk peptide library 2

Table S2. Interactions of coconut milk peptides with the key amino acids of various targets related to hyperglycemia and glycation 4

Figure S1. 2D representation of peptide 1 (MQIFVK) with the key amino acids of **(A)** α-glucosidase, **(B)** α-amylase, and **(C)** aldose reductase and 2D representation of peptide 2 (ADVFNPR) interacting with **(D)** key amino acids of α – glucosidase, **(E)** key amino acids of α – amylase**,** key amino acids of **(F)** aldose reductase, **(G)** Receptor for Advanced glycation end products (RAGE) 5

Figure S2. MD trajectories of control drugs-protein complexes. **(A-D)** Protein-ligand RMSD of the acarbose-α-glucosidase complex, acarbose-α-amylase, quercetin-aldose reductase, and protein-ligand RMSD of papaverine-RAGE, respectively **(E-H).** RMSF of α-glucosidase, α-amylase, aldose reductase, and RAGE, respectively **(I-L).** Ligand RMSF of acarbose concerning α-glucosidase, acarbose concerning α-amylase, quercetin concerning aldose reductase, and papaverine concerning RAGE, respectively **(M- P).** Ligand properties of reference drugs concerning α-glucosidase, α-amylase, aldose reductase, and RAGE, respectively 6

Figure S3. Chromatograms of the synthesised peptides. **(A)** peptide 1 (MQIFVK) **(B)** peptide 2 (ADVFNPR) 7

Figure S4. Mass spectra of the synthesised peptides. **(A)** peptide 1 (MQIFVK) **(B)** peptide 2 (ADVFNPR) 7

Figure S5. Lineweaver-Burk plot of substrate-dependent enzyme kinetics on inhibition of **(A)** α-glucosidase activity by peptide 2, **(B)** α - amylase activity by peptide 2 and **(C)** aldose reductase activity by peptide 2............ 8

Table S1. Outcomes of *insilico* analysis: Coconut milk peptide library

Coconut Milk Peptides Library										
**Protein description**	**Protein accession number**	**Peptide source**	**Peptide sequence**	**Stability**	**Length**	**Peptide ranker score**	**Bioactivity**	**Allergenicity**	**Toxicity**	**Solubility**
Uncharacterized protein OS=Cocos nucifera OX=13894 GN=COCNU_03G015580 PE=4 SV=1	A0A8K0I4T5	DSP-1	ISATGLGCKGGVGR	(-0.09 - stable)	14	0.577	Bioactive	Non-allergen	Non-toxin	Good water solubility
Uncharacterized protein OS=Cocos nucifera OX=13894 GN=COCNU_09G001010 PE=3 SV=1	A0A8K0IJ42	DSP-2	ETLLSFSDGPGRSAGAMR	24.95 - stable	18	0.51	Bioactive	Non-allergen	Non-toxin	Good water solubility
BRISC and BRCA1-A complex member 2 OS=Cocos nucifera OX=13894 GN=COCNU_08G008540 PE=3 SV=1	A0A8K0II65	DSP-2	VGQVWAGSKNGRYADR	(-19.36 - stable)	16	0.505	Bioactive	Non-allergen	Non-toxin	Good water solubility
Beta-1,2-xylosyltransferase XYXT1 OS=Cocos nucifera OX=13894 GN=COCNU_02G017800 PE=4 SV=1	A0A8K0I1A7	DSP-2	TYYGDPAVRMGLWYVGYK	26.01 - stable	18	0.523	Bioactive	Non-allergen	Non-toxin	Poor water solubility
non-specific serine/threonine protein kinase OS=Cocos nucifera OX=13894 GN=COCNU_01G005110 PE=3 SV=1	A0A8K0MU82	DSP-2	SGAAGVRLEGEELGR	20.38 - stable	15	0.581	Bioactive	Non-allergen	Non-toxin	Good water solubility
Abscisic acid 8'-hydroxylase 1 OS=Cocos nucifera OX=13894 GN=COCNU_10G010080 PE=3 SV=1	A0A8K0IN51	DSP-2	GAVADDLLGSFMESK	10.84 - stable	15	0.5964	Bioactive	Non-allergen	Non-toxin	Good water solubility
Putative Vesicle transport v-SNARE 13 OS=Cocos nucifera OX=13894 GN=COCNU_06G002140 PE=3 SV=1	A0A8K0IAK8	DSP-3	SGIDDAEAWIR	1.37 - stable	11	0.5892	Bioactive	Non-allergen	Non-toxin	Good water solubility
11S globulin isoform 2 OS=Cocos nucifera OX=13894 GN=COS-2 PE=2 SV=1	A0A0R7UCT6	DSP-2	ADVFNPR	(-18.44 - stable)	7	0.8847	Bioactive	Non-allergen	Non-toxin	Good water solubility
Zinc-finger homeodomain protein 6 OS=Cocos nucifera OX=13894 GN=COCNU_14G004720 PE=4 SV=1	A0A8K0NC03	DSP-3	MLAFAESVGWR	30.10 - stable	11	0.5381	Bioactive	Allergen	Non-toxin	Poor water solubility
Uncharacterized protein OS=Cocos nucifera OX=13894 GN=COCNU_01G001160 PE=4 SV=1	A0A8K0HSU0	DSP-3	KSELNMAFK	27.30 - stable	9	0.5983	Bioactive	Allergen	Non-toxin	Poor water solubility
NB-ARC domain-containing protein OS=Cocos nucifera OX=13894 GN=COCNU_06G010270 PE=4 SV=1	A0A8K0N2R7	DSP-3	LSDLGFENMK	9.00 - stable	10	0.5	Bioactive	Allergen	Non-toxin	Good water solubility
Lipid-A-disaccharide synthase OS=Novacetimonas cocois OX=1747507 GN=lpxB PE=3 SV=1	A0A365YRD6	DSPP-1	VASLSIPR	(-4.28 - stable)	8	0.6136	Bioactive	Allergen	Non-toxin	Poor water solubility
Ubiquitin-40S ribosomal protein S27a OS=Cocos nucifera OX=13894 GN=COCNU_01G000560 PE=3 SV=1	A0A8K0HSJ9	DSPP-3	MQIFVK	(-9.03 - stable)	6	0.8072	Bioactive	Non-allergen	Non-toxin	Good water solubility
Phosphoglycerate mutase (2,3-diphosphoglycerate-independent) OS=Cocos nucifera OX=13894 GN=COCNU_12G005940 PE=3 SV=1	A0A8K0IRS6	DSPP-3	SGQPLPDK	36.86 - stable	8	0.5343	Bioactive	Allergen	Non-toxin	Good water solubility
Restriction endonuclease subunit R OS=Novacetimonas cocois OX=1747507 GN=NJLHNGOC_11015 PE=4 SV=1	A0A365YT79	DSPP-3	DRTNPLNANFMK	(-24.57 - stable)	12	0.54	Bioactive	Non-allergen	Non-toxin	Good water solubility
Putative Elongation factor 1-alpha OS=Cocos nucifera OX=13894 GN=COCNU_04G002970 PE=3 SV=1	A0A8K0I545	DSPPT-1	IGGIGTVPVGR	32.17 - stable	11	0.501	Bioactive	Allergen	Non-toxin	Poor water solubility
Oleosin 1 OS=Cocos nucifera OX=13894 GN=COCNU_03G014580 PE=3 SV=1	A0A8K0MZD6	DSP-2	HPPGADRLDAARAAMAR	11.88 - stable	17	0.6484	Bioactive	Allergen	Non-toxin	Good water solubility
Histone H4 OS=Cocos nucifera OX=13894 GN=COCNU_07G002830 PE=3 SV=1	A0A8K0N4K0	DSP-1	ISGLIYEETR	34.06 - stable	16	0.556	Non-bioactive	Allergen	Non-toxin	Good water solubility
Uncharacterized protein OS=Novacetimonas cocois OX=1747507 GN=NJLHNGOC_06330 PE=4 SV=1	A0A365YX23	DSP-2	AATGEAPAAQ	31.49 - stable	10	0.0956	Non-bioactive	Allergen	Non-toxin	Good water solubility
Glycine-rich protein-like OS=Cocos nucifera OX=13894 GN=COCNU_01G012420 PE=4 SV=1	A0A8K0HVU7	DSP-2	TDEVGVHDEK	0.51 - stable	10	0.0425	Non-bioactive	Allergen	Non-toxin	Good water solubility
Actin OS=Oryctes rhinoceros OX=72550 PE=2 SV=1	A0A5C0C9N0	DSP-2	SYELPDGQVITIGNER	1.97 - stable	16	0.2061	Non-bioactive	Non-allergen	Non-toxin	Good water solubility
Serine/threonine-protein kinase EDR1 OS=Cocos nucifera OX=13894 GN=COCNU_02G000880 PE=4 SV=1	A0A8K0HX85	DSP-2	QINLSSAPYEADSTQTK	29.54 - stable	17	0.1173	Non-bioactive	Non-allergen	Non-toxin	Good water solubility
Uncharacterized protein OS=Cocos nucifera OX=13894 GN=COCNU_15G004100 PE=4 SV=1	A0A8K0NCW1	DSP-2	KVETTDGKSAHAR	(-10.36 - stable)	13	0.0665	Non-bioactive	Non-allergen	Non-toxin	Good water solubility
Ribosomal protein L19/L19e domain-containing protein OS=Cocos nucifera OX=13894 GN=COCNU_01G002000 PE=4 SV=1	A0A8K0HT59	DSP-2	VSDGKGSNR	(-9.98 - stable)	9	0.1645	Non-bioactive	Non-allergen	Non-toxin	Good water solubility
Putative Phosphoglucan phosphatase LSF1, chloroplastic OS=Cocos nucifera OX=13894 GN=COCNU_06G007280 PE=4 SV=1	A0A8K0N2Z0	DSP-2	RGGNAEKSR	25.13 - stable	9	0.1285	Non-bioactive	Non-allergen	Non-toxin	Good water solubility
Gibberellin 20-oxidase-like protein OS=Cocos nucifera OX=13894 GN=COCNU_10G008630 PE=3 SV=1	A0A8K0IMY4	DSP-2	FRENSVEGR	8.89 - stable	9	0.1837	Non-bioactive	Allergen	Non-toxin	Good water solubility
Protein LNK2 OS=Cocos nucifera OX=13894 GN=COCNU_04G007090 PE=4 SV=1	A0A8K0N069	DSP-2	LPGAEANTNPIDR	(-8.59 - stable)	13	0.2547	Non-bioactive	Non-allergen	Non-toxin	Good water solubility
DNA ligase OS=Cocos nucifera OX=13894 GN=COCNU_16G001250 PE=3 SV=1	A0A8K0NDS7	DSP-2	TLDKDATYEPSK	27.91 - stable	12	0.0845	Non-bioactive	Non-allergen	Non-toxin	Good water solubility
Heat shock 70 kDa protein 17 OS=Cocos nucifera OX=13894 GN=COCNU_05G009480 PE=4 SV=1	A0A8K0N204	DSP-2	DAVISVPPYFGQAER	35.01 - stable	15	0.2926	Non-bioactive	Non-allergen	Non-toxin	Good water solubility

Coconut Milk Peptides Library										
**Protein description**	**Protein accession number**	**Peptide source**	**Peptide sequence**	**Stability**	**Length**	**Peptide ranker score**	**Bioactivity**	**Allergenicity**	**Toxicity**	**Solubility**
Pumilio domain-containing protein C6G9.14 OS=Cocos nucifera OX=13894 GN=COCNU_13G002960 PE=4 SV=1	A0A8K0ISW6	DSP-2	TIGSGADNAGR	(-6.35 - stable)	11	0.1919	Non-bioactive	Allergen	Non-toxin	Good water solubility
B3 domain-containing protein OS=Cocos nucifera OX=13894 GN=COCNU_02G010120 PE=4 SV=1	A0A8K0HZJ7	DSP-3	RGAPETTPARTMTR	35.03 - stable	14	0.2031	Non-bioactive	Non-allergen	Non-toxin	Good water solubility
Basic leucine zipper 19 OS=Cocos nucifera OX=13894 GN=COCNU_09G004450 PE=4 SV=1	A0A8K0IJI0	DSP-3	RPSGNREAVR	39.03 - stable	10	0.1868	Non-bioactive	Non-allergen	Non-toxin	Good water solubility
Phosphoglycerate mutase-like protein AT74 OS=Cocos nucifera OX=13894 GN=COCNU_12G001460 PE=4 SV=1	A0A8K0IR86	DSP-3	MTPKQHNGER	30.91 - stable	10	0.1413	Non-bioactive	Allergen	Non-toxin	Good water solubility
Tubulin alpha chain OS=Cocos nucifera OX=13894 GN=COCNU_06G019320 PE=3 SV=1	A0A8K0ID58	DSP-3	AVFVDLEPTVIDEVR	12.15 - stable	15	0.1507	Non-bioactive	Non-allergen	Non-toxin	Good water solubility
Zinc finger CCCH domain-containing protein 32 OS=Cocos nucifera OX=13894 GN=COCNU_07G014120 PE=4 SV=1	A0A8K0IG26	DSP-3	REPASGNAAICSKGDNNNQK	16.50 - stable	20	0.4223	Non-bioactive	Non-allergen	Non-toxin	Good water solubility
Vicilin-like antimicrobial peptides 2-2 OS=Cocos nucifera OX=13894 GN=COCNU_06G015680 PE=3 SV=1	A0A8K0N3Q7	DSP-3	EVDEVLNAPR	5.69 - stable	10	0.1401	Non-bioactive	Non-allergen	Non-toxin	Good water solubility
Protein terminal ear1 OS=Cocos nucifera OX=13894 GN=COCNU_08G003660 PE=4 SV=1	A0A8K0N612	DSP-3	SHHGKNGGGEAR	(-4.79 - stable)	12	0.2609	Non-bioactive	Non-allergen	Non-toxin	Good water solubility
Enhancer of polycomb-like protein OS=Cocos nucifera OX=13894 GN=COCNU_03G016160 PE=3 SV=1	A0A8K0I451	DSP-3	TESLLTNDGLER	28.74 - stable	12	0.0724	Non-bioactive	Allergen	Non-toxin	Good water solubility
Uncharacterized protein OS=Cocos nucifera OX=13894 GN=COCNU_07G001990 PE=4 SV=1	A0A8K0IHK3	DSP-3	QSALTEVDRGSDIPEHGR	25.41 - stable	18	0.1635	Non-bioactive	Non-allergen	Non-toxin	Good water solubility
CW-type domain-containing protein OS=Cocos nucifera OX=13894 GN=COCNU_16G003210 PE=4 SV=1	A0A8K0NDY2	DSP-3	KSSTGRMDK	11.42 - stable	9	0.1488	Non-bioactive	Non-allergen	Non-toxin	Good water solubility
TetR family transcriptional regulator OS=Novacetimonas cocois OX=1747507 GN=NJLHNGOC_06140 PE=4 SV=1	A0A365YXJ4	DSPP-1	DAGLSLDVAR	9 - stable	10	0.2685	Non-bioactive	Allergen	Non-toxin	Good water solubility
NAC domain-containing protein OS=Cocos nucifera OX=13894 GN=COCNU_12G005790 PE=4 SV=1	A0A8K0IRR6	DSPP-1	TLSGandVPNGLK	(-0.58 - stable)	13	0.2679	Non-bioactive	Non-allergen	Non-toxin	Good water solubility
Enhanced ethylene response protein 5 OS=Cocos nucifera OX=13894 GN=COCNU_11G008510 PE=4 SV=1	A0A8K0N9I2	DSPP-2	MALHLSMGEAHR	6.09 - stable	12	0.2261	Non-bioactive	Non-allergen	Non-toxin	Poor water solubility
Protein CELLULOSE SYNTHASE INTERACTIVE 1 OS=Cocos nucifera OX=13894 GN=COCNU_05G006420 PE=4 SV=1	A0A8K0I8N2	DSPP-2	LLGPGNEASIR	1.37 - stable	11	0.2407	Non-bioactive	Non-allergen	Non-toxin	Good water solubility
5-methyltetrahydropteroyltriglutamate--homocysteine S- methyltransferase OS=Cocos nucifera OX=13894 GN=COCNU_03G000470 PE=3 SV=1	A0A8K0MXV5	DSPP-2	EVIAELK	(-3.56 - stable)	7	0.0674	Non-bioactive	Allergen	Non-toxin	Good water solubility
Mediator of RNA polymerase II transcription subunit 12 OS=Cocos nucifera OX=13894 GN=COCNU_10G008500 PE=3 SV=1	A0A8K0IM04	DSPP-3	VSNCTGVFSAPTDDGISVR	(26.32 - stable)	19	0.2539	Non-bioactive	Allergen	Non-toxin	Good water solubility
Putative epidermal growth factor receptor substrate 15-like 1 OS=Cocos nucifera OX=13894 GN=COCNU_08G009480 PE=4 SV=1	A0A8K0N723	DSPP-3	SEIVLSGSHER	36.39 - stable	11	0.1228	Non-bioactive	Allergen	Non-toxin	Good water solubility
Protein SAAL1 OS=Cocos nucifera OX=13894 GN=COCNU_06G013400 PE=4 SV=1	A0A8K0N3C9	DSPP-3	LGEDLKNSNAHK	24.52 - stable	12	0.1829	Non-bioactive	Non-allergen	Non-toxin	Good water solubility
inorganic diphosphatase OS=Cocos nucifera OX=13894 GN=COCNU_05G010360 PE=3 SV=1	A0A8K0N1S3	DSPPT-1	AIGLMPMIDQGEKDDK	22.11 - stable	16	0.1637	Non-bioactive	Allergen	Non-toxin	Good water solubility

Table S2. Interactions of coconut milk peptides with the key amino acids of various targets related to hyperglycemia and glycation

Name of the peptides	Name of the target	Key amino acids with the type of interactions	Mode of action of peptides	References
**Peptide 1**	α - glucosidase	Asp 242, Asp 352, Gly 277 (hydrogen bond), and Asp 69 (salt bridge)	Inhibition	**[49]**
	α - amylase	Lys 200, Asp 300, and Arg 303 (hydrogen bond), Glu 233 (salt bridge)	Inhibition	**[47]**
	aldose reductase	Tyr 48, Lys 77, His 110, Glu 120, and Ser 302 (hydrogen bond), Trp 20 (Pi-Pi stacking), and Asp 43 (salt bridge)	Inhibition	**[49]**
**Peptide 2**	α - glucosidase	Arg 315 (hydrogen bond), Pro 312 (hydrogen bond), Gly 161 (hydrogen bond), Asp 352 (salt bridge)	Inhibition	**[49]**
	α - amylase	Glu 233 (hydrogen bond), Asn 300 (hydrogen bond, salt bridge), Asn 301 (hydrogen bond), Gln 63 (hydrogen bond)	Inhibition	**[49]**
	aldose reductase	Trp 20, Tyr 48, Gln 49, Glu 120 (hydrogen bond), Glu 53 (salt bridge)	Inhibition	**[49]**
	RAGE	Arg 57 (hydrogen bond, salt bridge), Arg 179 (hydrogen bond, salt bridge), Glu 94, Val 117, Thr 154, Ser 211, Gly 213 (hydrogen bond)	Inhibition	**[71]**

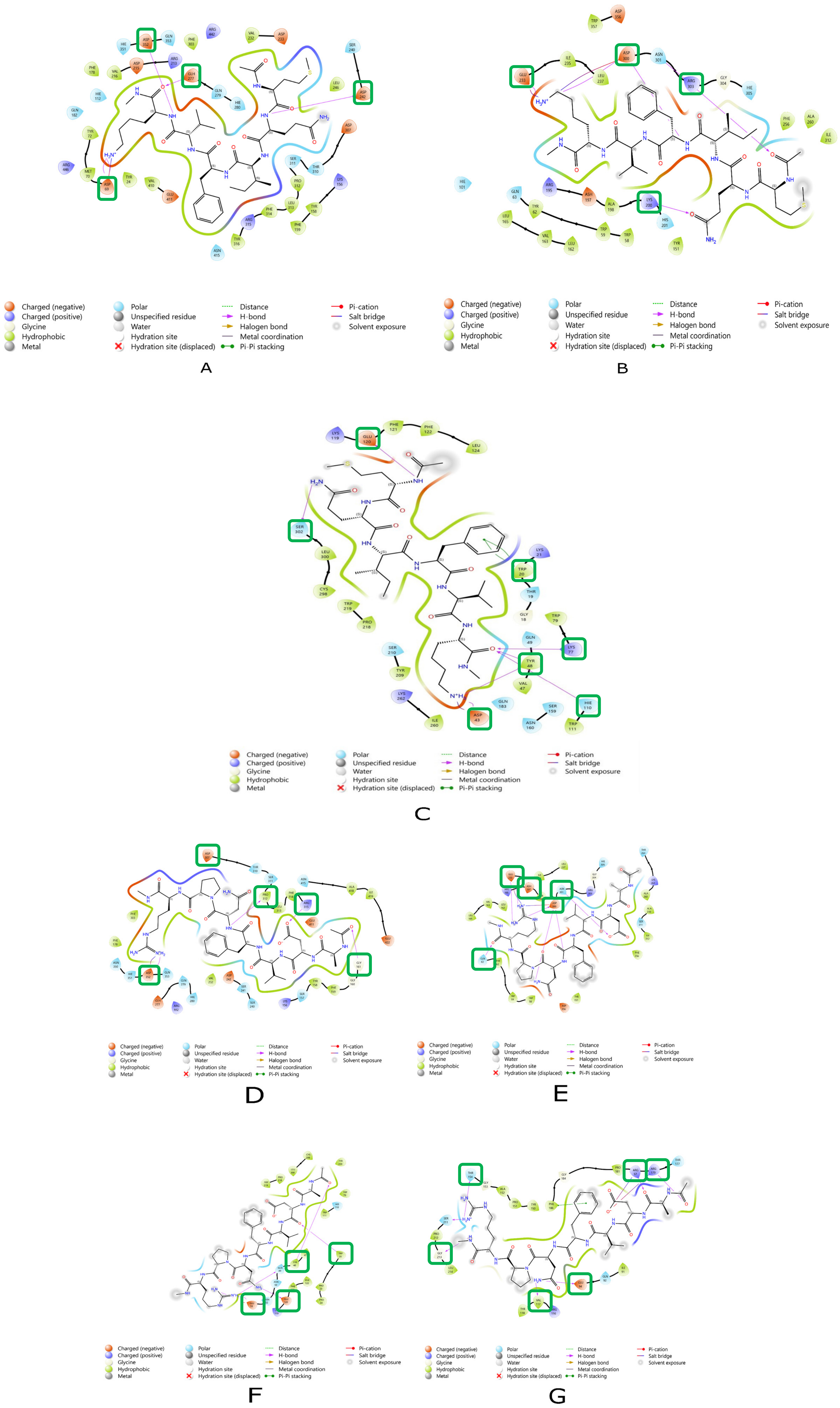

Figure S1. 2D representation of peptide 1 (MQIFVK) with the key amino acids of **(A)** α-glucosidase, **(B)** α-amylase, and **(C)** aldose reductase and 2D representation of peptide 2 (ADVFNPR) interacting with **(D)** key amino acids of α – glucosidase, **(E)** key amino acids of α – amylase**,** key amino acids of **(F)** aldose reductase, **(G)** Receptor for Advanced glycation end products (RAGE)

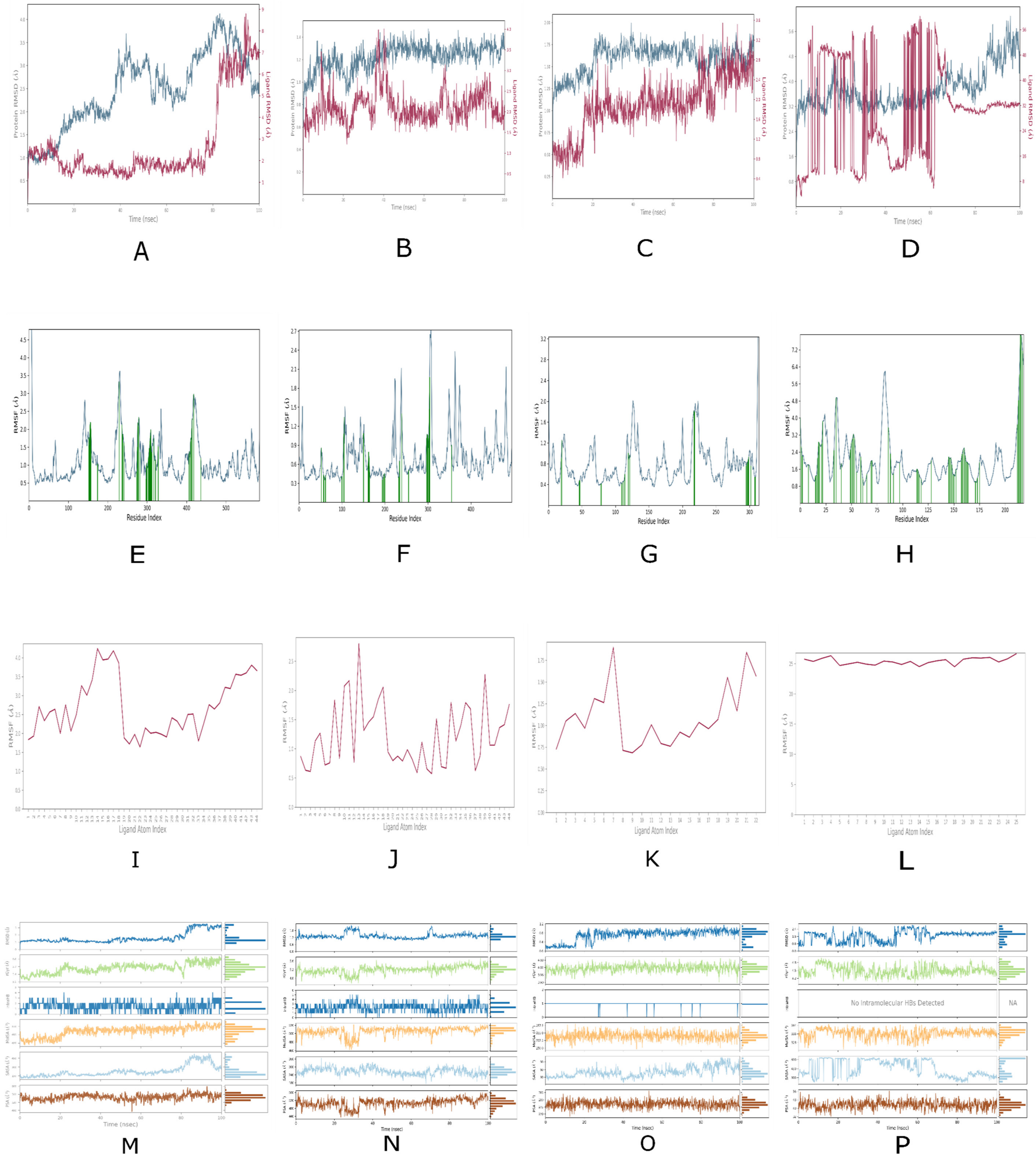

Figure S2. MD trajectories of control drugs-protein complexes. **(A-D)** Protein-ligand RMSD of the acarbose-α-glucosidase complex, acarbose-α-amylase, quercetin-aldose reductase, and protein-ligand RMSD of papaverine-RAGE, respectively **(E-H).** RMSF of α-glucosidase, α-amylase, aldose reductase, and RAGE, respectively **(I-L).** Ligand RMSF of acarbose concerning α-glucosidase, acarbose concerning α-amylase, quercetin concerning aldose reductase, and papaverine concerning RAGE, respectively **(M- P).** Ligand properties of reference drugs concerning α-glucosidase, α-amylase, aldose reductase, and RAGE, respectively.

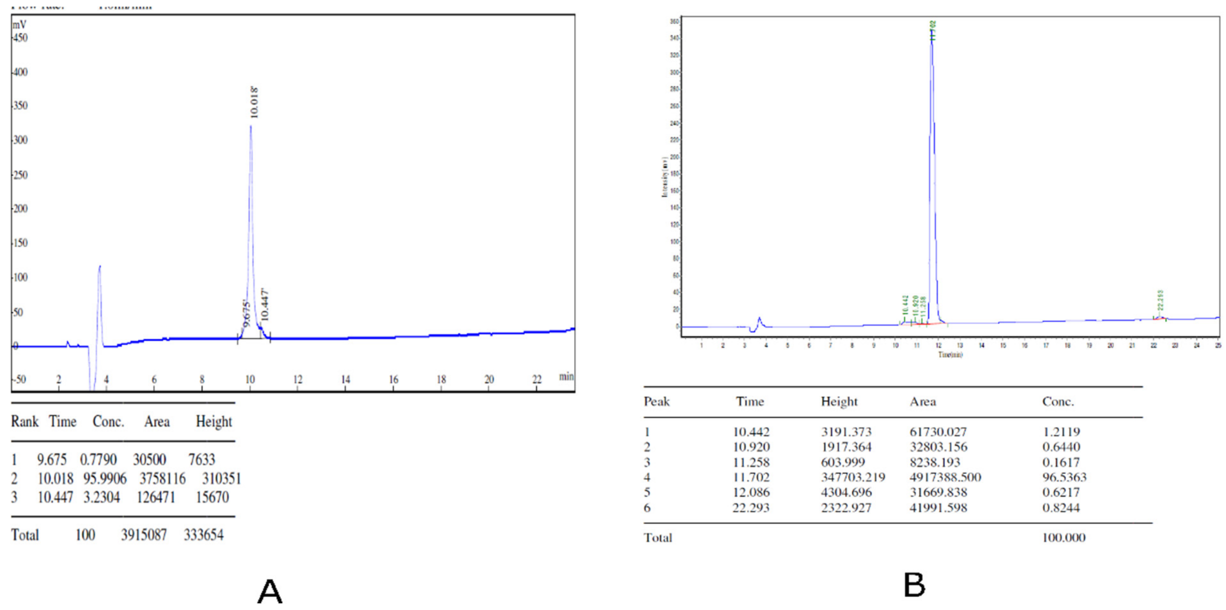

Figure S3. Chromatograms of the synthesised peptides. **(A)** peptide 1 (MQIFVK) **(B)** peptide 2 (ADVFNPR)

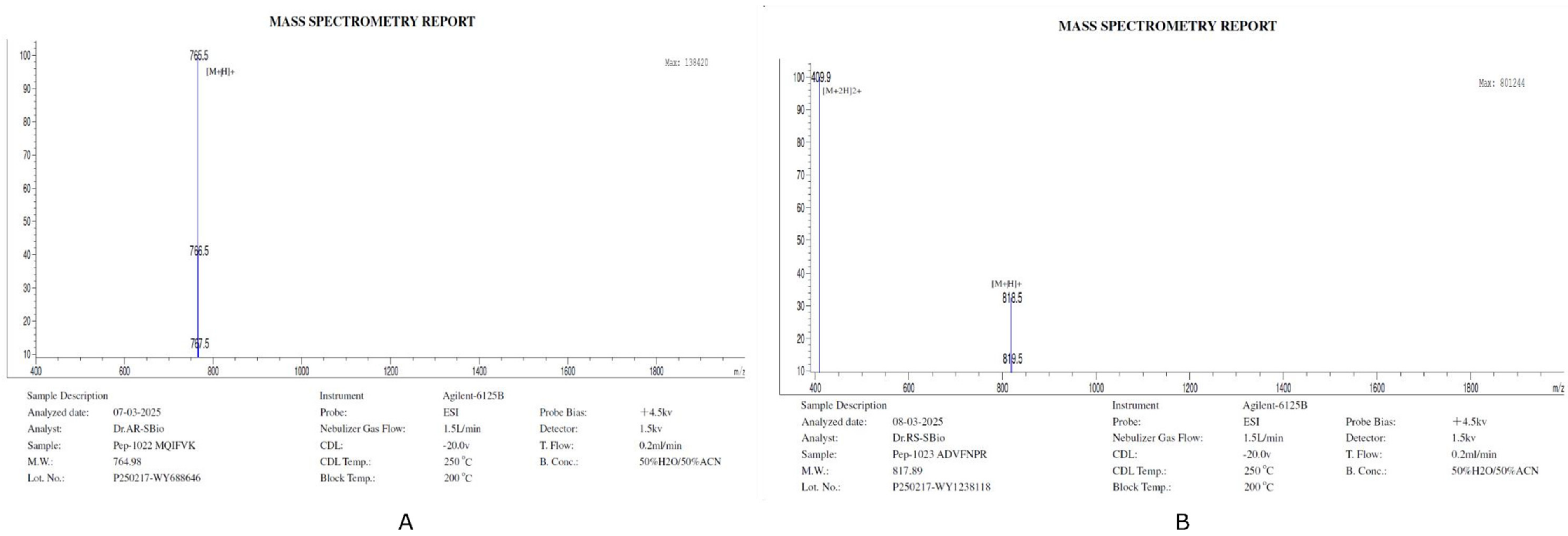

Figure S4. Mass spectra of the synthesised peptides. **(A)** peptide 1 (MQIFVK) **(B)** peptide 2 (ADVFNPR)

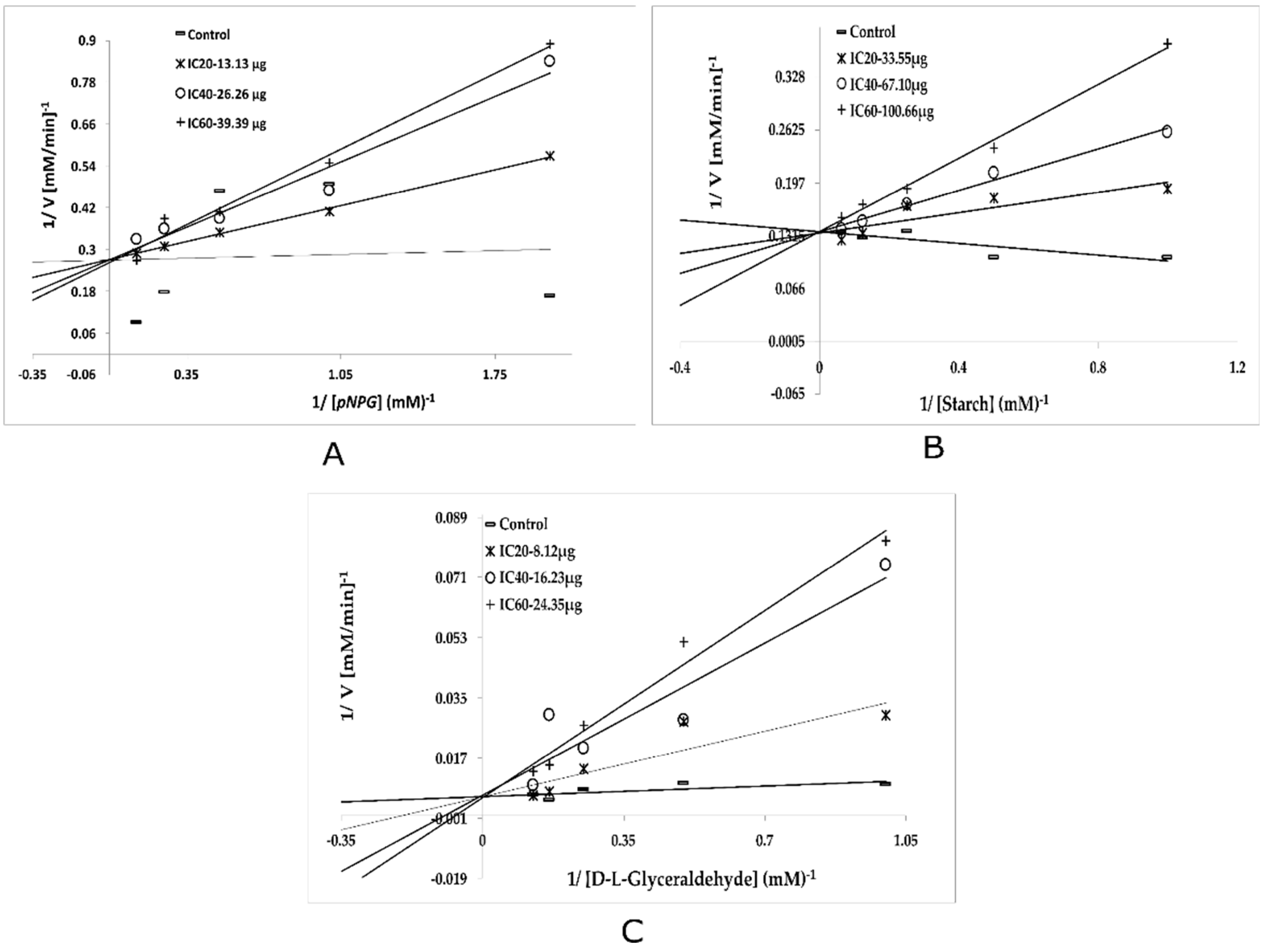

Figure S5. Lineweaver-Burk plot of substrate-dependent enzyme kinetics on inhibition of **(A)** α-glucosidase activity by peptide 2, **(B)** α - amylase activity by peptide 2 and **(C)** aldose reductase activity by peptide 2
